# First report on clinical aspects, blood profiles, bacterial isolation, antimicrobial susceptibility, and histopathology in canine pyometra in Thailand

**DOI:** 10.14202/vetworld.2022.1804-1813

**Published:** 2022-07-26

**Authors:** Niyada Lansubsakul, Kaitkanoke Sirinarumitr, Theerapol Sirinarumitr, Kanjana Imsilp, Podjana Wattananit, Sasi Supanrung, Chunsumon Limmanont

**Affiliations:** 1 Department of Anatomy, Faculty of Veterinary Medicine, Kasetsart University, Bangkok, Thailand; 2 Department of Companion Animal Clinical Sciences, Faculty of Veterinary Medicine, Kasetsart University, Bangkok, Thailand; 3 Theriogenology Center, Kasetsart University Veterinary Teaching Hospital, Bangkok, Thailand; 4 Department of Pathology, Faculty of Veterinary Medicine, Kasetsart University, Bangkok, Thailand; 5 Department of Pharmacology, Faculty of Veterinary Medicine, Kasetsart University, Bangkok, Thailand; 6 Department of Clinical Science and Public Health, Faculty of Veterinary Science, Mahidol University, Nakhon Pathom, Thailand

**Keywords:** antimicrobial susceptibility, bacteria, canine, histopathology, pyometra

## Abstract

**Background and Aim:**

Canine pyometra, either the closed (closed pyometra [CP]) or open (open pyometra [OP]) cervix type, is a frequent uterine disease in intact old age bitches. Therefore, early diagnosis and appropriate medical and surgical treatments are crucial to avoid the life-threatening condition in these bitches. This study aimed to investigate clinical alterations, blood parameters, causative bacteria, antimicrobial susceptibility, and uterine histopathology obtained during aseptic surgical treatment on bitches with pyometra.

**Materials and Methods:**

Sixty bitches of various breeds and ages with presumptive pyometra diagnoses were included in the study. The diagnoses were based on history, clinical examination, blood parameters, radiography, and ultrasonography. All pyometra bitches were ovariohysterectomized as an emergency surgical treatment. In addition, uterine content and tissues were submitted for bacterial isolation, antimicrobial susceptibility, and uterine histopathological analysis.

**Results:**

Except for abdominal CP distention, no specific clinical signs were linked to the pyometra type. The mean values of total white blood cell count (WBC) and plasma protein were predominantly raised in pyometra bitches regarding hematological parameters. Leukocytosis was found in both types; however, the WBC in CP was markedly higher than in OP. The mean value of blood urea nitrogen increased in the CP group. *Klebsiella pneumoniae* and *Escherichia coli* were the most frequent causative bacteria isolated in CP and OP, respectively. All isolated bacteria were 100% susceptible to imipenem, meropenem, and carbapenem. Marbofloxacin was the second most effective drug against isolated bacteria from both groups. Uncomplicated cystic endometrial hyperplasia (CEH) was not presented in the CP group. CEH and chronic endometritis (type IV), the most severe uterine histopathological changes, were discovered in the CP and OP.

**Conclusion:**

The CP and OP groups presented leukocytosis, increased plasma protein, and CEH and chronic endometritis. Depression, abdominal distention, and enlarged uterine size were the major characteristics of the CP group. Furthermore, abdominal distension is presented in other abnormalities in clinical practices, providing a differential diagnosis. Drugs in the carbapenem group were the most effective against isolated bacteria; however, they are not routinely used due to bacterial resistance concerns. Thus, marbofloxacin was recommended as an alternative medical treatment because it is convenient to manage by both oral and injection routes.

## Introduction

Canine pyometra is a frequent uterine disease in intact mid- to old-aged bitches [1, 2]. Purulent content accumulates in the uterine lumen with systemic illnesses [3, 4]. Hormonal and bacterial factors are fundamental for disease pathogenesis, which manifests as potentially life-threatening [1, 3]. During the estrous cycle, the high estrogen level in the estrous phase plays an indirect role because it enhances the endometrial response to progesterone. In the luteal phase (diestrus), the progesterone level increases, leading to increased secretion from the endometrial glands, increased proliferation of the endometrium, and reduced myometrium contraction and cervix [4, 5]. The repeated estrus cycle twice a year in a bitch is a predisposing cause of cystic endometrium hyperplasia (CEH). Factors associated with bacterial infection, and their receptor expression, may lead to enhanced bacterial attachment to the endometrium [4, 6]. Pyometra pathogenesis is linked with changes in the expression of oxytocin and sex steroid receptors in the canine reproductive tract [7]. *Escherichia coli* is the most common bacterium found in the uterine content of pyometra bitches [4]. Its virulence, antigen, and cytotoxic necrotizing factors are associated with more severe pathological conditions. Other pathogenic bacteria, including *Klebsiella* spp., *Streptococcus* spp., *Staphylococcus* spp., and *Pseudomonas* spp., have also been identified as causative pyometra agents [4]. An antibiogram is vital for pre-and post-operative care medical treatment of pyometra bitches. Empirical antimicrobial treatment before receiving the susceptibility result is performed following previous reports and veterinarian experience [3]. Selecting an appropriate antimicrobial agent for each bitch with pyometra can provide more effective treatment and help prevent antimicrobial-resistant problems. The causative bacteria in a recent study from 55 pyometra bitches revealed that *E. coli* was the most isolated microorganism (63.6%) followed by *Streptococcus* spp. (10.9%) and *Pseudomonas* spp. (9.1%). The top three highest susceptibilities of 62 pathogen isolates were imipenem (95.2%), gentamicin (85.5%), and amikacin (80.6%) [3]. Pyometra is usually diagnosed from 4 weeks to 4 months post-estrus period [2]. Pyometra bitches commonly present with lethargy, anorexia, fever, polyuria (PU), and polydipsia (PD), with or without vaginal discharge and abdominal enlargement. The diagnosis is based on history, clinical signs, estrus cycle stage, hormonal use, physical examination, blood profiles, abdominal radiography, and/or ultrasonography [2, 8]. Early diagnosis and appropriate medical treatment are necessary to avoid life-threatening conditions in bitches. An ovariohysterectomy (OVH) and a specific antimicrobial drug are conventional therapies for pyometra bitches [3]. Pyometra classification is based on the status of the cervix as closed (closed pyometra [CP], without vaginal discharge) or open (open pyometra [OP], with vaginal discharge). The pathophysiology of either type is unknown [7]. Another classification is based on uterine histopathology (e.g., CEH and endometritis). Canine pyometra varies from other uterine pathological conditions (e.g., hydrometra, mucometra, and chronic endometritis hyperplasia complex) [2]. Histopathology is recommended for the final diagnosis of pyometra [8, 9].

No published literature on canine pyometra relates clinical aspects, blood profiles, bacterial isolation, antimicrobial susceptibility, and histopathology to pyometra types (CP and OP). The availability of this information will be significant in helping veterinarians treat pyometra patients. Moreover, they can help with better or earlier diagnosis and, consequently, the antibiotic selection before receiving susceptibility results.

This study aimed to investigate statistically related clinical alterations, blood parameters, causative bacteria, antimicrobial susceptibility, and uterine histopathology to pyometra types.

## Materials and Methods

### Ethical approval and informed consent

All procedures were performed in accordance with the guidelines approved by the Kasetsart University Institutional Animal Care and Use Committee (approval number #ACKU61-VET-026) and Ethical Review Board of the Office of National Research Council of Thailand, Institute of Animals for Scientific Purpose Development. A signed consent form was obtained from each owner.

### Study period and location

The study period was 21 months, from September 2017 to July 2019. The investigation was performed as a prospective study. The blood, uterine content, and uterine tissue, including all medical data, were collected from pyometra bitches diagnosed and immediately operated on at the Kasetsart University Veterinary Teaching Hospital (KUVTH), Bangkok, Thailand. All laboratory procedures were performed at the Kasetsart University Veterinary Diagnostic Center, Bangkhen Campus, Thailand.

### Animal and data collection from pyometra bitches

Sixty female dogs of various breeds and ages were included in this study, and all had a presumptive pyometra diagnosis. All the dogs were treated as emergency cases at KUVTH. Tentative pyometra was diagnosed based on history, clinical signs, examinations, blood profiles, radiography, and/or ultrasonography. Abnormal signs in the uterus were detected using ultrasonography using a 13 MHz broadband linear transducer (GE™, Fairfield, CT, USA) and an experienced veterinary radiologist. They consisted of an enlarged uterus, endometrial cysts, irregular endometrium surface, and uterine enlargement filled by echoic-to-hyperechoic material of the uterus. In addition, a signalment, history, and clinical signs (e.g., breed, age, body weight, estrous period, contraceptive drug use, mental status, appetite, vaginal discharge feature, vomit, rectal temperature, abdominal distension, abdominal pain, and laboratory parameters) were tested and recorded. Bitches were classified into four groups based on body size of 1–10, 10.01–25, 25.01–40, and >40 kg, described as small, medium, large, and giant breeds, respectively. Obese bitches were evaluated following World Small Animal Veterinary Association guidelines [10].

Pyrexia was evaluated as adapted from another study, and bitches were classified as the following: Without fever (100.5°F ≤ temperature (T) < 102.5°F), moderate fever (102.5°F ≤ T < 104.9°F), and high fever (T ≥ 104.9°F) [9]. The life stage of each bitch was classified as presented in Table-1 [11]. Bitches with pyometra were diagnosed based on animal history (intact, old age, and use of contraception), clinical findings (depression, anorexia, PU/PD, abdominal distension, abdominal pain, and with/without vaginal discharge), and hematology (leukocytosis and leukopenia). Radiography and/or ultrasonography demonstrated an enlarged, fluid-filled uterus (with anechoic or hypoechoic). A final diagnosis of pyometra was confirmed post-OVH by uterine content and histopathological analysis.

**Table 1:** Life stage classification of bitches [11].

Size breed	Life stage	Age
Small	Puppy	3 months–12 months
	Adult	>12 months–7 years
	Senior	>7 years
Medium	Puppy	3 months–12 months
	Adult	>12 months–5 years
	Senior	>5 years
Large	Puppy	3 months–18 months
	Adult	>18 months–5 years
	Senior	>5 years
Giant	Puppy	3 months–18 months
	Adult	>18 months–5 years
	Senior	>5 years

#### Blood sampling and analysis

Before treatment, 5 mL of blood sample was collected from each animal’s cephalic vein. The samples were contained in two types of evacuated tubes. The first was coated with ethylenediaminetetraacetic acid to determine the complete blood count. The second sample was kept in a tube without any anticoagulant for the evaluation of serum biochemistry – blood urea nitrogen (BUN), creatinine (Cr), and alanine aminotransferase (ALT). The samples were centrifuged at 12,000× *g* for 15 min at 25°C and then analyzed within 2 h after collection to separate serum. Complete blood count and serum biochemistry were performed following the manufacturers’ instructions for the automated veterinary hematology analyzer (Sysmex XN-1000V™, Guangzhou, China) and the automated clinical chemistry analyzer (ILab Taurus™, Milan, Italy).

#### Collections of uterine content and tissues

The diameters of each uterine horn and body were measured and recorded immediately after the removal of each uterus. First, a uterine horn was randomly selected for content and tissue collection. A random sample was then taken from the contents of each uterine horn for bacterial isolation under aseptic conditions using sterile blades to stab and subsequently sterilize cotton swabs. Consequently, a sample measuring 0.5 × 0.5 cm (length × width) was taken from the same side of each horn for later histopathological analysis.

#### Bacterial isolates and antimicrobial susceptibility

Uterine content samples were placed in the transport media. Bacterial isolates were kept in sheep blood and MacConkey agar (Merck Millipore, MA, USA) at 37°C for 24 h with 5% CO_2_. Bacterial identification was performed following colony characteristics and biochemical tests [12]. In addition, antimicrobial susceptibility was determined using the modified Kirby–Bauer method. Briefly, a bacterial colony was suspended and cultured in Mueller–Hinton agar either with or without 5% blood, and antimicrobial disks were placed by a dispenser. Following overnight incubation, any inhibition zone was measured and compared to published zone diameter interpretive standards for veterinary pathogens [12]. In total, 15 antimicrobial agents in eight classes were used in this study (Table-2).

**Table 2:** Antimicrobial drugs used for susceptibility test of bacterial species isolated from uterine contents in pyometra bitches.

No.	Class	Name of antibiotic
1	Penicillins (β-lactams)	Amoxicillin
2		Amoxicillin-clavulanic acid
3	Cephalosporins (β-lactams)	Cephalexin
4		Ceftriaxone
5	Fluoroquinolones	Ciprofloxacin
6		Enrofloxacin
7		Marbofloxacin
8		Norfloxacin
9	Lincosamide	Clindamycin
10	Macrolide	Azithromycin
11	Carbapenems	Imipenem
12		Meropenem
13	Tetracycline	Doxycycline
14	Aminoglycoside	Gentamicin
15	Sulfonamide- trimethoprim	Sulfamethoxazole- trimethoprim

#### Histopathological analysis

**Figure-1:**
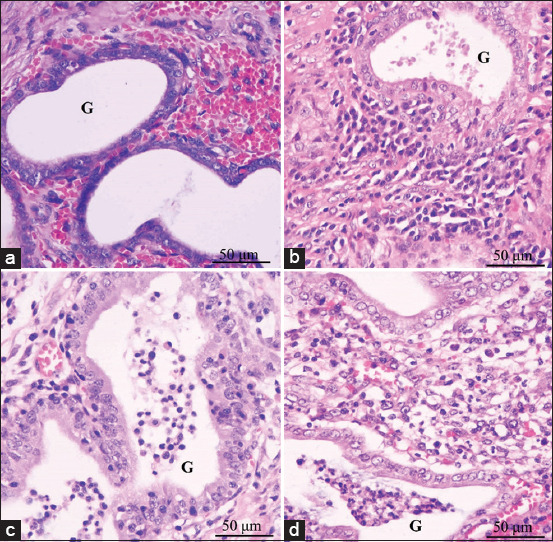
Hematoxylin and eosin-stained histological images of transverse uterine horn fragment showing endometrial layer of uterus, with uterine gland (G). Histopathological classification into uncomplicated cystic endometrial hyperplasia (CEH) (a), CEH and plasma cell infiltrated (b), CEH and acute endometritis (c), and CEH and chronic endometritis (d), with all 100×.

Fresh uterine tissue was fixed in 10% paraformaldehyde for 24 h. The specimens were processed by embedding in paraffin, sectioning, and staining with hematoxylin and eosin. The morphological structures of the pyometra were observed under a light microscope (Olympus™, Tokyo, Japan). Table-3 and Figure-1 show histopathological lesions, and types of CEH in CP and OP were examined by a Thai board-certified pathologist, according to Dow [13].

**Table 3:** Histopathological types and lesions of CEH from canine uterine tissues.

Type of CEH	Histopathological results for uterus	Brief description of characteristics
Type I	Uncomplicated CEH	CEH without inflammation, no clinical signs
Type II	CEH and plasma cell infiltrate	CEH with the slightly enlarged uterus. Plasma cells infiltration in uterus without damage to endometrium. Neutrophilia without clinical signs
Type III	CEH and acute endometritis	Acute endometritis with neutrophil infiltration, congestion, and edema of the endometrium. Neutrophilia with clinical signs such as enlarged abdomen and may present vaginal discharge. The discharges are various, presented as reddish-brown to yellowish-green color
Type IV	CEH and chronic endometritis	Chronic endometritis with cystic and atrophic endometrium. Thickening endometrium and neutrophilia. A large amount of discharge in uterus in closed cervix pyometra. The severe clinical signs depend on white blood cells, distended abdomen and damage to other abdominal organs. May or may not present vaginal discharge

CEH=Cystic endometrial hyperplasia

### Statistical analysis

Using the Kolmogorov–Smirnov test, the normal distributions of age, body weight, uterus size, blood profile, and blood chemistry data were analyzed. Age, hemoglobin, packed cell volume (PCV), plasma protein, and the size of the uterine body between the CP and OP groups were compared using unpair Student’s *t*-test analysis because their distributions were normal. In contrast, differences in body weight, size of uterine horns, other blood profiles, and blood chemistry data between the two groups were analyzed using the Mann–Whitney *U*-test because normal distributions were not confirmed.

The Chi-square test was performed to explore the relationship of each clinical sign, blood profile, and blood chemistry level to the pyometra type. A multinomial logistic regression model was used to test the association of groups of all parameters with the pyometra type. All statistical tests were performed using the SPSS Statistics v.18.0 software (SPSS Inc., Chicago, IL, USA). The significant difference was set at p < 0.05. Bacterial isolates, antimicrobial susceptibility, and histopathology were presented based on percentage.

## Results

### Animal, clinical signs, and blood profiles from pyometra bitches

Most pyometra bitches (66.04%, 35/53) had purulent discharge, were senior (68.33%, 41/60), small in breed size (76.67%, 46/60), and obese (55.00%, 33/60). The most common clinical signs in the pyometra bitches were depression (71.67%, 43/60), anorexia (66.67%, 40/60), and no fever (81.67%, 49/60).

Of the bitches, 19 and 41 were diagnosed as CP and OP, respectively. For the OP bitches, the main type of vaginal discharge was purulent (63.41%), with other types listed in Table-4. Most uterine content in CP bitches was purulent (75%), and the remainder was bloody and purulent (25%). Only 12 from 19 bitches with CP were noted in the uterine content type.

**Table 4:** Percentages of parameters in total pyometra bitches (n = 60) and within CP (CP, n = 19) and OP (OP, n = 41) groups.

Parameter	Percentage of total pyometra bitches			Percentage within individual group		p-value*
	
	%CP (n)	%OP (n)	%CP + %OP (n)	%CP (n)	%OP (n)	
Uterine content	n = 53			n = 12	n = 41	
Purulent	16.98 (9)	49.06 (26)	66.04 (35)	75.00 ( 9)	63.41 (30)	0.82
Bloody with purulent	5.66 (3)	24.53 (13)	30.19 (16)	25.00 (3)	31.71 (13)	
Bloody with mucoid	0.00 (0)	1.89 (1)	1.89 (1)	0.00 (0)	2.44 (1)	
Purulent with mucoid	0.00 (0)	1.89 (1)	1.89 (1)	0.00 (0)	2.44 (1)	
Life stage	n = 60			n=19	n=41	
Puppy	0.00 (0)	1.67 (1)	1.67 (1)	0.00 (0)	2.44 (1)	0.61
Adult	11.67 (7)	18.33 (11)	30.00 (18)	36.84 (7)	26.83 (11)	
Senior	20.00 (12)	48.33 (29)	68.33 (41)	63.16 (12)	70.73 (29)	
Bitch size	n = 60			n=19	n=41	
Small	26.67 (16)	50.00 (30)	76.67 (46)	84.21 (16)	73.17 (30)	0.64
Medium	1.67 (1)	10.00 (6)	11.67 (7)	5.26 (1)	14.63 (6)	
Large	3.33 (2)	6.67 (4)	10.00 (6)	10.53 (2)	9.76 (4)	
Giant	0.00 (0)	1.67 (1)	1.67 (1)	0.00 (0)	2.44 (1)	
Obesity	11.67 (7)	43.33 (26)	55.00 (33)	36.84 (7)	63.41 (26)	0.05
Contraceptive drug use	1.82 (1/55)	5.45 (3/55)	7.27 (4/55)	5.88 (1/17)	7.89 (3/38)	0.79
Clinical signs	n = 60			n=19	n=41	
Depression	25.00 (15)	46.67 (28)	71.67 (43)	78.95 (15)	68.29 (28)	0.40
Anorexia	21.67 (13)	45.00 (27)	66.67 (40)	68.42 (13)	65.85 (27)	0.84
Vomit	13.33 (8)	20.00 (12)	33.33 (20)	42.11 (8)	29.27 (12)	0.33
Abdominal pain	11.67 (7)	18.33 (11)	41.67 (25)	36.84 (14)	26.83 (11)	0.43
Abdominal distention	23.33 (14)	15.00 (9)	38.33 (23)	73.68 (14)	21.95 (19)	0.00
PU/PD	11.67 (7)	15.00 (9)	26.67 (16)	36.84 (7)	21.95 (9)	0.22
Fever	n = 60			n=19	n=41	
No fever	25.00 (15)	56.67 (34)	81.67 (49)	78.95 (15)	82.93 (34)	0.92
Moderate fever	5.00 (3)	8.33 (5)	13.33 (8)	15.79 (3)	12.20 (5)	
High fever	1.67 (1)	3.33 (2)	5.00 (3)	5.26 (1)	4.88 (2)	

Data of uterine content of seven CP bitches are missing.

* Crosstab or Chi-square test (association to pyometra type), PU/PD=Polyuria/polydipsia, CP=Closed pyometra, OP=Open pyometra

The percentages of each signalment and history data (life stage, bitch size, and birth control) within each pyometra type are presented in Table-4. CP and OP were mostly presented in senior bitches (63.16% and 70.73%, respectively), and only one OP bitch was as a puppy (2.44%). Most pyometra bitches were from the small breed size (CP, 84.21%; OP, 73.17%). Bitches in the OP group (63.41%) were notably obese (p = 0.054), compared with those in the CP group (36.84%). Only a few CP (5.88%) and OP (7.89%) bitches had a history of contraceptive drug reception.

The percentages of clinical findings (e.g., discharge type, anorexia, depression, vomit, abdominal pain, abdominal distention, PU/PD, and fever within each pyometra type) are presented in Table-4. The main clinical signs presented in CP bitches were depression (78.95%), abdominal distension (73.68%), and anorexia (68.42%). The OP bitches presented major clinical signs of depression (68.29%) and anorexia (65.85%). The clinical sign of abdominal distension was significantly (p < 0.01) greater in CP than in OP bitches. Most CP (78.95%) and OP (82.93%) bitches had no fever. Only a few cases of CP (5.26%) and OP (4.88%) bitches had a high fever.

The percentages of blood parameter alterations within each pyometra type are shown in Table-5. Most CP (57.89%, 11/19) and OP (65.85%, 27/41) bitches had a normal PCV level. Most CP (89.47%, 17/19) and OP (65.85%, 27/41) bitches presented with leukocytosis. All pyometra bitches (CP + OP) predominantly had normal PCV (63.33%, 38/60) and ALT (90.74%, 49/54) levels but had leukocytosis (73.33%, 44/60). Furthermore, one and three OP bitches developed severe anemia (1.67%, 1/60) and leukopenia (5.00%, 3/60), respectively. Furthermore, pyometra bitches presented with thrombocytopenia (53.34%, 32/60) and azotemia (20.33%, 12/59).

**Table 5:** Percentages of hematological parameters and blood biochemistry levels in total pyometra bitches (n = 60) and within CP (CP, n = 19) and OP (OP, n = 41) groups.

Parameter	Percentage of total pyometra bitches			Percentage within individual group		p-value*
	
	%CP (n)	%OP (n)	%CP + %OP (n)	%OP (n)	%CP (n)	
Anemia	n = 60			n = 19	n = 41	
No anemia	18.33 (11)	45 (27)	63.33 (38)	57.89 (11)	65.85 (27)	0.27
Mild anemia (30% ≤ PCV<35%)	10.00 (6)	8.33 (5)	18.33 (11)	31.58 (6)	12.2 (5)	
Moderate anemia (20% ≤ PCV < 30%)	3.33 (2)	13.33 (8)	16.67 (10)	10.53 (2)	19.51 (8)	
Severe anemia severe (PCV < 20%)	0.00 (0)	1.67 (1)	1.67 (1)	0.00 (0)	2.44 (1)	
WBC	n = 60			n = 19	n = 41	
Normal WBC	3.33 (2)	18.33 (11)	21.67 (13)	10.53 (2)	26.83 (11)	0.14
Leukocytosis (WBC > 17 × 10^3^/cumm)	28.33 (17)	45.00 (27)	73.33 (44)	89.47 (17)	65.85 (27)	
Leukopenia (WBC < 6 × 10^3^/cumm)	0.00 (0)	5.00 (3)	5.00 (3)	0.00 (0)	7.32 (3)	
Thrombocytopenia (PLT < 200 × 10^3^/μL)	21.67 (13)	31.67 (19)	53.33 (32)	68.42 (13)	46.34 (19)	0.11
Azotemia (BUN > 26 mg% and/or Cr > 1.3 mg%)	10.17 (6/59)	10.17 (6/59)	20.33 (12/59)	31.58 (6/19)	15.00 (6/40)	0.14
Elevated ALT (ALT > 70 IU/L)	1.79 (1/54)	7.14 (4/54)	9.26 (5/54)	5.56 (1/17)	11.11 (4/34)	0.50

* Cross tab or Chi-square test (association to pyometra type), PCV=Packed cell volume, WBC=Total white blood cell count, PLT=Platelets cell count, BUN=Blood urea nitrogen, Cr=Creatinine, ALT=Alanine aminotransferase, CP=Closed pyometra, OP=Open pyometra

The mean ± SD values of age, body weight, and size of the uterus are presented in Table-6. The sizes of the left and right horns of CP bitches were significantly larger (p < 0.05) than for the OP group. However, age, body weight, and the body of the uterus showed no significant difference. Table-7 summarizes the mean ± SD values of hematology and blood chemistry levels. All pyometra bitches had elevated white blood cell (WBC) and plasma protein levels; however, the WBC in the CP group was also significantly (p < 0.05) higher than for the OP group (Table-7).

**Table 6:** Mean ± SD of age, bodyweight, and size of uterus of closed (n = 19) and open (n = 41) pyometra groups.

Parameter (unit)	Closed pyometra (range)	Open pyometra (range)
Age (years)	8.51 ± 2.44 (4.00–1.00)	8.78 ± 3.40 (1.00–15.00)
Body weight (kg)	7.56 ± 6.88 (1.80–27.50)	11.66 ± 12.41 (1.55–59.00)
Size of uterus (cm)		
Left horn*	3.95 ± 1.84^a^ (1.15–8.14)	2.47 ± 1.24^b^ (0.67–6.35)
Right horn*	3.69 ± 1.55^a^ (1.05–6.37)	2.28 ± 1.01^b^ (0.91–4.95)
Body	2.20 ± 1.38 (0.60–5.05)	1.89 ± 1.13 (0.57–7.33)

Values with different lowercase superscripts within the same row indicate significant (p < 0.05) differences between closed and open pyometra groups.

* Significant difference between closed and open pyometra groups of these parameters determined using Mann–Whitney U-test. SD=Standard deviation

**Table 7:** Mean ± SD of hematology parameters and blood biochemistry levels of closed (n=19) and open (n=41) pyometra groups.

Parameter (unit)	Closed pyometra (range)	Open pyometra (range)	Reference range
Hematology			
Hb (g%)	12.42 ± 2.12 (8.96–16.40)	12.74 ± 3.00 (5.52–17.90)	10.00–18.00
RBC (×10^6^/cumm)	5.39 ± 0.95 (3.90–7.35)	5.54 ± 1.21 (2.35–7.60)	5.00–9.00
PCV (%)	35.44 ± 5.80 (26.00–46.60)	36.58 ± 8.14 (16.30–49.70)	35.00–55.00
WBC* (×10^3^/cumm)	45.51 ± 26.71a (11.50–118.00)	24.05 ± 14.81b (4.80–66.70)	6.00–17.00
PLT (×10^3^/µL)	212.51 ± 211.05 (1.49–648.00)	203.44 ± 126.13 (5.64–648.00)	200.00–900.00
PP (g%)	8.39 ± 1.08 (7.00–10.20)	8.08 ± 1.32 (4.80–11.40)	6.00–7.50
Blood chemistry profiles			
BUN** (mg%)	32.79 ± 40.66 (6.00–167.00)	24.08 ± 27.58 (5.00–161.00)	10.00–26.00
Cr** (mg%)	1.28 ± 0.74 (0.42–2.77)	1.04 ± 0.55 (0.40–2.79)	0.50–1.30
ALT** (IU/L)	42.11 ± 93.80 (11.00–416.00)	40.14 ± 24.22 (13.00–131.00)	6.00–70.00

Values with different lowercase superscripts within the same row indicate significant (p<0.05) differences between closed and open pyometra groups.

* Significant difference between closed and open pyometra groups of these parameters determined using Mann–Whitney U-test.

** Only serum BUN values of 57 pyometra bitches (19 CP bitches and 38 OP bitches), serum Cr values of 59 pyometra bitches (19 CP bitches and 40 OP bitches), and serum ALT values of 54 pyometra bitches (18 CP bitches and 36 OP bitches) were evaluated and recorded in the medical report. Hb=Hemoglobin, RBC=Red blood cell count, PCV=Packed cell volume, WBC=Total white blood cell count, PLT=Platelet cell count, PP=Plasma protein, BUN=Blood urea nitrogen, Cr=Creatinine, ALT=Alanine aminotransferase, SD=Standard deviation

No clinical signs (e.g., anorexia, depression, vomit, abdominal pain, fever, and PU/PD) were significantly associated with the pyometra type except for abdominal distention (p < 0.01), which was greater in CP than in OP bitches. In addition, the status of blood chemistry (e.g., anemia, platelet count, azotemia, and ALT level) was not significantly different between the pyometra types (Table-5). The best-fit model of parameters that significantly (R^2^ = 0.271, p < 0.05) presented any association with the pyometra type was for the combination of abdominal distention and obesity.

### Bacterial profiles and antimicrobial susceptibility

**Figure-2:**
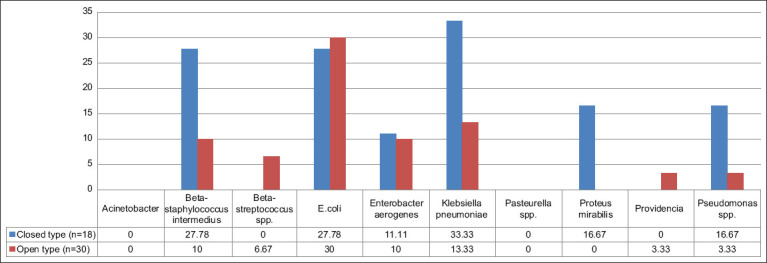
Percentage by bacterial species from aerobic bacterial culture from uterine content of closed (n = 18) and open (n = 30) pyometra groups.

**Figure-3:**
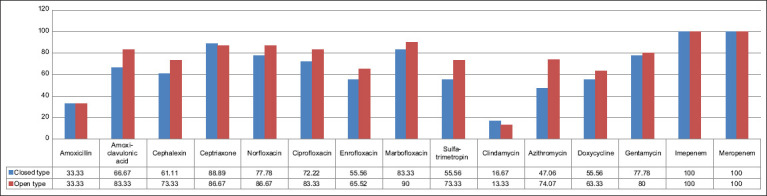
Percentages of bacteria isolated from uterine content culture of closed (n = 18) and open (n = 30) pyometra groups susceptible to antimicrobial drugs.

No bacterial growth in either group was noted; the percentages of single and coinfection bacterial infections from aerobic bacteria isolated from CP and OP are presented in Table-8. The numbers of bacteria identified and susceptible to antimicrobial drugs resulting from single and coinfection samples are presented in Figures-2 and 3, respectively.

**Table 8:** Percentages of aerobic bacterial infections from uterine content of closed (n=19) and open (n = 40) pyometra groups.

Aerobic bacterial infection	% closed pyometra (n)	% open pyometra* (n)
No bacterial growth	5.26% (1)	25.00% (10)
Single infection	63.16% (12)	57.50% (23)
Coinfection	31.58% (6)	17.50% (7)

* One open pyometra bitch sample was not submitted for aerobic bacterial culture

### Histopathological findings

The number of dogs with a specific type of CEH/total of CP cases and the number of dogs with a specific type of CEH/total of OP cases are presented as percentages (Table-9). Most histopathological findings of uterine tissues from closed and OP were CEH and chronic endometritis (52.94% and 55.26%, respectively).

**Table 9:** Number of dogs with a specific type of CEH/total of closed pyometra cases (n = 17) and number of dogs with a specific type of CEH/total of open pyometra cases (n = 38) are presented as percentages.

Type of CEH	Histopathological results for uterus	%closed pyometra (n)	%open pyometra (n)
Type I	Uncomplicated CEH	0.00% (0)	5.26% (2)
Type II	CEH and plasma cell infiltrate	11.76% (2)	2.63% (1)
Type III	CEH and acute endometritis	35.29% (6)	36.84% (14)
Type IV	CEH and chronic endometritis	52.94% (9)	55.26% (21)

CEH=Cystic endometrial hyperplasia

## Discussion

In this study, 68.33% (41/60) of the bitches were diagnosed with OP, which was more common than CP. This result corroborated with another study [14]. The bitches with OP illness are easily detectable through vaginal discharge by the owner. In this study, small breed dogs contributed to the highest number of pyometra cases (76.66%; 46/60), followed by medium (11.67%; 7/60), large (10%; 6/60), and giant (0.02%; 1/60) breeds. These results conformed to another study regarding the incidence of pyometra, and the potential risk factor was the size of bitches [15]. It may be possible that the small breed is more popular in the city for several reasons (e.g., limited space, social value, and economic reasons). Nonetheless, the present study did not correlate with the bitch size based on body weight and pyometra type. Cross-breed bitches were most commonly affected in this study, followed by Pomeranians and Chihuahuas. Other studies in the UK and Sweden found Blue Mastiffs, Golden Retrievers, Dogue de Bordeaux, Rottweiler, and Cavalier King Charles Spaniels to be the most common pyometra bitches [16, 17]. Thus, this could be due to the preferred breeds of people in various countries.

Bitches with pyometra mainly found in this study were senior (>8 years). This result was similar to previous reports that pyometra normally affects middle- to old-aged bitches, with the median age ranging from 6.50 to 9.36 years [15–19]. Older bitches risk having pyometra because they have more estrous cycles than younger ones. The repeated estrous cycle causes a repeatedly elevated estrogen level that results in endometrium proliferation and extends the period of uterine cervix opening. This period developed the prolongate interval of progesterone dominance pending the diestrus period. Progesterone activates the proliferation of the endometrium with enhanced secretion from the uterine gland and reduced myometrium contraction, which causes the cervix to close. This increases uterine susceptibility to infection because of the invasion by bacteria into the luminal uterus, finally causing pyometra. In addition, progesterone promotes the progress of uterine receptors that permit bacterial adhesion. For embryo implantation, glycocalyx expression on the apical surface of the uterine endometrium is required. These sugar residues are discovered in the uterine glandular epithelium and adhere to the bacterial targets in CEH. These outcomes improve when the estrous cycle repeats in older bitches [20]. The immune response and bacterial infection resistance decrease as age increases [21]. Nevertheless, the present study did not correlate pyometra type and bitch age. The different results could be prejudiced because CP is frequently undetectable by dog owners or undiagnosed due to the lack of vaginal discharge.

Fever is an inconsistent condition usually related to bacterial infection, uterine inflammation, and septicemia [11, 18]. In this study, pyometra did not influence fever and agreed with previous studies [18, 22, 23]. The influence of pyometra on other clinical signs (e.g., anorexia, depression, vomit, PU, PD, and abdominal pain) varied, which was similar to previous studies [18, 24, 25]. The results showed that abdominal distention and uterus size were significantly associated with pyometra types. The uterus was markedly enlarged in CP cases. Therefore, this could be the effect of an activated uterine gland, intraluminal exudate accumulation [26], and a closed cervix. In clinical practices that should conduct differential diagnoses, abdominal distension is also presented in other abnormalities (e.g., hepatomegaly, splenomegaly, ascites, and visceral mass). Pyometra bitches were predominantly obese at 56.67%. Thus, obesity may facilitate the risk of infection related to immune system dysregulation and decreased cell-mediated immune responses [27].

The most frequent hematological abnormality in pyometra bitches is leukocytosis [14]. The CP bitches indicated a significantly greater magnitude of leukocytosis than OP bitches, similar to another report [14]. The increase in WBC reflects the higher inflammatory responses induced by the disease [28]. Moreover, CP causes more accumulation of numerous pathogens, inducing progressive inflammation and consequently leukocytosis. In this study, leukopenia was also observed in obese OP patients (3/60). Leukopenia increased the risk of peritonitis 18-fold and enhanced the risk of prolonged hospitalization by 3.5-fold, resulting from endotoxemia-induced bone marrow suppression [28]. The incidence of anemia and normal hematocrit in suspected pyometra bitches was revealed, similar to prior studies [14, 24].

Anemia sometimes occurs following the chronic illness stage or the systemic inflammatory response. Furthermore, red blood cell diapedesis in the uterus may result from the toxic suppression of erythropoiesis [29]. Concurrent diseases (e.g., anaplasmosis and ehrlichiosis) were detected in the current study, which caused severe blood abnormalities. These infections are tick-borne diseases and are frequently found in Thailand. Therefore, additional tests should be performed.

Nevertheless, the average biochemistry blood profiles (Cr, BUN, and ALT) were within the normal range and did not differ between the CP and OP groups. These results follow other observations [14, 24]. For example, rapid detection before the liver and kidney is injured due to systemic inflammatory responses and multiple organ dysfunctions make the treatment successful [30].

Pyometra is a bacterial infection of the uterus that causes uterine inflammation [5, 31]. Aerobic bacteria are mostly bacteria isolated from pyometra samples [11]. In this study, predominantly isolated bacteria from uterine content in OP and CP groups were *E. coli* and *K. pneumoniae*, respectively. The results of *K. pneumoniae* in CP bitches were inconsistent with other reports in which *E. coli* was the most common infectious agent [3, 16, 32]. Various uterine bioenvironments between CP and OP could play a major role in microbial isolates [32]. The study showed no bacterial growth in 5.26% and 25.00% of the CP and OP groups, respectively. A primary reason was that some pyometra bitches received empirical treatment before bacterial culture [6, 23, 29]. Another reason was that anaerobic bacteria were excluded from the bacterial culture panel. A report on *Hafnia paralvei* (formerly *Hafnia alvei*), a Gram-negative, mobile, rod-shaped facultative anaerobe isolated from canine emphysematous pyometra in a Labrador Retriever bitch, was noted [33].

Meropenem and imipenem were the best antimicrobial drugs for both CP (100%) and OP (100%), which was similar to another report [3]. However, the carbapenem group is not the first drug of choice in clinical practices because of bacterial resistance concerns [34]. The second and third choices of antimicrobial agents were marbofloxacin and gentamicin, respectively. The nephrotoxicity of gentamicin limits its clinical uses because the renal function of pyometra bitches may be altered through dehydration and/or toxicity [3]. Marbofloxacin, which is convenient to manage by both oral and injection routes, is the best alternative.

Numerous changes have been reported in the uterine histomorphology in pyometra bitches [13]. Pyometra severity was associated with histopathological alterations. CEH and chronic endometritis (type IV) were the most common histopathological types discovered in both CP and OP. A few CEHs (type I) samples were found in OP (5.26%) but not in CP bitches. The CEH (type I) was rarely detected because neither clinical nor subclinical signs were noted [30].

## Conclusion

The bitches in this study who were at higher risk of pyometra were >8 years old. Another risk factor for both pyometra types was the small breed. The disease’s main signs were depression and anorexia. The abdomen and uterus of CP patients were substantially larger. The mean values of the WBC predominantly increased in both pyometra types. However, the CP bitches showed a statistically greater magnitude of leukocytosis than the OP bitches. The average biochemistry blood profiles (Cr, BUN, and ALT) were within the normal range and did not differ between the CP and OP groups. Predominantly isolated bacteria from uterine content in OP and CP groups were *E. coli* and *K. pneumoniae*, respectively. Meropenem and imipenem were the best antimicrobial drugs for both CP (100%) and OP (100%). However, the carbapenem group is not the first drug of choice in clinical practices because of bacterial resistance concerns. Marbofloxacin, which is convenient to manage by oral and injection routes, is the best alternative. CEH and chronic endometritis (type IV) were the most common histopathological types found in both CP and OP.

In summary, early diagnosis and appropriate treatments for pyometra bitches are essential for favorable consequences and successful treatment. This study’s limitation was the lack of complete data (e.g., insufficient information from the owners, estrous cycle stage, vaginal cytology, contraception history, previous antimicrobial use, cost and availability of antimicrobial agents, and after-surgery follow-up). Further study of bacterial resistance genes and the bacterial polymerase chain reaction will be important for successful diagnosis and treatment. Encouragement of OVH in bitches to owners will help prevent pyometra.

## Authors’ Contributions

NL and CL: Conducted the literature review, performed the study, interpreted the data, and drafted the manuscript. KS: Conceived the idea and designed the study. NL and CL: Drafted and reviewed the manuscript. TS: Performed histopathological study. KI: Verified the analytical methods, supervised the study, and reviewed the manuscript. PW: Verified the analytical methods, interpreted the data, and reviewed the manuscript. SS: Developed the theory, designed the study, and performed the study. All authors have read and approved the final manuscript.
